# The regulation of oncogenic Ras/ERK signalling by dual-specificity mitogen activated protein kinase phosphatases (MKPs)

**DOI:** 10.1016/j.semcdb.2016.01.009

**Published:** 2016-02

**Authors:** Andrew M. Kidger, Stephen M. Keyse

**Affiliations:** CR-UK Stress Response Laboratory, Division of Cancer Research, Jacqui Wood Cancer Centre, Ninewells Hospital & Medical School, Dundee DD1 9SY, UK

**Keywords:** MAP kinase, Extracellular signal-regulated kinase, MAP kinase phosphatase, Dual-specificity phosphatase, *DUSP5*, *DUSP6*/MKP-3, Ras oncogene

## Abstract

Dual-specificity MAP kinase (MAPK) phosphatases (MKPs or DUSPs) are well-established negative regulators of MAPK signalling in mammalian cells and tissues. By virtue of their differential subcellular localisation and ability to specifically recognise, dephosphorylate and inactivate different MAPK isoforms, they are key spatiotemporal regulators of pathway activity. Furthermore, as they are transcriptionally regulated as downstream targets of MAPK signalling they can either act as classical negative feedback regulators or mediate cross talk between distinct MAPK pathways. Because MAPKs and particularly Ras/ERK signalling are implicated in cancer initiation and development, the observation that MKPs are abnormally regulated in human tumours has been interpreted as evidence that these enzymes can either suppress or promote carcinogenesis. However, definitive evidence of such roles has been lacking. Here we review recent work based on the use of mouse models, biochemical studies and clinical data that demonstrate key roles for MKPs in modulating the oncogenic potential of Ras/ERK signalling and also indicate that these enzymes may play a role in the response of tumours to certain anticancer drugs. Overall, this work reinforces the importance of negative regulatory mechanisms in modulating the activity of oncogenic MAPK signalling and indicates that MKPs may provide novel targets for therapeutic intervention in cancer.

## 1 Dual-specificity MAP kinase phosphatases: spatiotemporal regulators of MAP kinase signalling

**Fig. 1:**
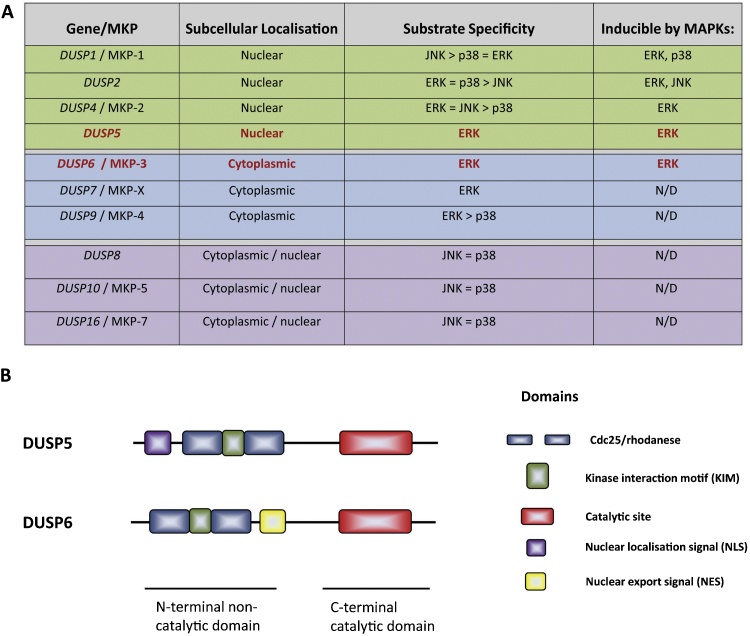
(A) List of the mammalian dual specificity MAP kinase phosphatases broken down into three groups by sequence similarity, subcellular localisation and substrate specificity. The ERK-specific phosphatases *DUSP5* and *DUSP6*/MKP-3, which are the main subject of the experimental work covered in this review are highlighted in red. ND, not determined. (B) Schematics showing the domain structures of *DUSP5* and *DUSP6*/MKP-3 highlighting the disposition of the kinase interaction motif (KIM) and localisation signals located within the N-terminal non catalytic domain and the catalytic site within the C-terminal domain.

A subfamily of 10 catalytically active dual-specificity protein phosphatases are dedicated to the task of negatively regulating the mammalian mitogen-activated protein kinases (MAPKs) by dephosphorylating both tyrosine and threonine residues of the signature T-X-Y motif located within the activation loop of the kinase [1], [2], [3]. These MAPK phosphatases (MKPs or DUSPs) can be broken down into three subgroups based on sequence homology, subcellular localisation and substrate specificity. There are four inducible nuclear MKPs; *DUSP1*/MKP-1, *DUSP2*, *DUSP4*/MKP-2 and *DUSP5*. Three MKPs; *DUSP6*/MKP-3, *DUSP7* and *DUSP9*/MKP-4 are both cytoplasmic and ERK selective while a further three MKPs; *DUSP8*, *DUSP1*0/MKP-5 and *DUSP16*/MKP-7 preferentially inactivate the stress-activated c-Jun amino-terminal kinase (JNK) and p38 MAPKs (Fig. 1A). These enzymes share a highly conserved structure comprising of a non-catalytic N-terminal domain and a C-terminal catalytic domain. The latter contains the highly conserved protein tyrosine phosphatase (PTPase) active site sequence (I/V)HCXAGXXR(S/T/G). The N-terminal domain contains a conserved modular sequence known as the kinase interaction motif (KIM), which mediates differential recognition and binding of MAPK substrates and also harbours either nuclear localisation (NLS) or export (NES) signals, which determine subcellular localisation (Fig. 1B) [4]. Interestingly, in a subset of MKPs the conformation of the active site in the absence of substrate is not optimal for catalysis. However, when a MAPK substrate is engaged via the KIM, this causes an allosteric rearrangement of the active site residues within the C-terminal catalytic domain resulting in catalytic activation, a process thought to underpin greater substrate selectivity [5], [6]. Finally, The observation that the KIM-mediated binding of MKPs to their cognate substrates by interaction with the conserved MAPK common docking (CD) domain does not require phosphorylation and activation of the MAPK itself has led to the idea that MKPs, by sequestering inactive MAPKs within either the nucleus or the cytoplasm, may regulate the spatial localisation as well as the duration and magnitude of signalling [7].

Many of the genes that encode MKPs are themselves highly inducible and in many cases the activity of one or more MAPK pathways is responsible for transcriptional up regulation of these enzymes. Thus, individual MKPs can act as classical negative feedback regulators of pathway activity, but can also mediate crosstalk between distinct MAPK modules. The fact that MAPK signalling and in particular the activity of the Ras–extracellular signal-regulated kinase (Ras/ERK) pathway is often abnormally activated in human cancers, suggests that MKPs may also be regulated as a result of the oncogenic activation of MAPK signalling. This idea is reinforced by several studies demonstrating that negative feedback control of Ras/ERK signalling by MKPs may play an important role in determining the biological outcome of signalling when upstream components of this pathway such as receptor tyrosine kinases (RTKs), Ras isoforms or Braf are mutated and activated [8], [9]. This idea is also supported by numerous observations of either increased or decreased MKP expression in malignant disease, suggesting that these enzymes might play some role in cancer initiation and/or progression [10], [11]. However, despite the large number of reported studies of MKP dysregulation the majority of both *in vitro* and tumour studies rely heavily on the overexpression of MKPs and/or correlations between MKP expression levels and clinical stage/outcome in relatively small patient cohorts. There is therefore a need for more defined genetic studies of MKP function in validated mouse models of cancer in which Ras/ERK signalling is implicated, coupled with more systematic analyses of MKP expression in large clinical cohorts before firm conclusions can be reached as to the role and significance of MKPs in malignant disease.

Here we review a number of recent studies using a variety of approaches including pharmacological and genetic manipulation of MKP expression or activity, which point towards specific roles for individual MKPs in either the suppression or promotion of carcinogenesis in human malignancies in which abnormal activation of the Ras/ERK pathway is an established driver of cancer development. Furthermore, there is also an increasing body of evidence suggesting that MKPs may play an important role in determining the outcome of either conventional or novel anti-cancer drugs that target components of this pathway. Overall, these studies indicate that MKPs play complex and context dependent roles in both tumour initiation and development and in drug responses, but there are also indications that under certain circumstances the manipulation of MKP activity or expression might be used to gain therapeutic advantage in cancer treatment.

## 2 ERK-specific MKPs and cancer

### 2.1 DUSP6/MKP-3: a potential tumour suppressor

*DUSP6*/MKP-3 was the first MKP with absolute substrate specificity for ERK, as opposed to either JNK or p38, to be characterised [12], [13]. The enzyme is cytoplasmic and this, coupled with its high ERK binding affinity, led to the demonstration that it can act as both a regulator of and cytoplasmic anchor for ERKs [14]. *DUSP6*/MKP-3 expression is growth factor inducible and this phosphatase plays a key role in the regulation of fibroblast growth factor (FGF) -dependent ERK activation during early embryonic development [15], [16]. Despite early confusion as to which Ras effector pathway signals to the *DUSP6*/MKP-3 promoter, with both phosphatidylinositide 3-kinase (PI3K) and ERK signalling implicated in its regulation, it is now clear that *DUSP6*/MKP-3 is an ERK target gene and that it acts as a classical negative feedback regulator of ERK activity, both in a developmental context and in primary and cancer cell lines [16], [17], [18]. Furthermore, *DUSP6*/MKP-3 is one of a small group of genes that is consistently upregulated in response to elevated ERK signalling in cancer cells harbouring activating mutations in either Ras or Braf, where it is presumed to restrain oncogenic ERK signalling [19], [20], [21].

Perhaps the most compelling evidence for a role for *DUSP6*/MKP-3 as a tumour suppressor has come from studies of pancreatic cancer in which 90% of tumours contain activating mutations in *Kras*. Although there is no evidence of mutation within the *DUSP6* gene, mRNA expression levels are consistently lower in pancreatic cancer cell lines when compared to immortalised normal pancreatic ductal cells [22], [23]. Analysis of *DUSP6*/MKP-3 expression in pancreatic tumours revealed that protein levels were increased in early pancreatic intraepithelial neoplasia (PanIN), but then reduced in more advanced invasive or poorly differentiated tumours, a result confirmed in a more extensive study [22], [24]. At the molecular level the loss of *DUSP6*/MKP-3 expression in both pancreatic tumour cell lines and in advanced pancreatic cancer was correlated with the methylation of CpG sequences in intron 1 of the *DUSP6* gene, suggesting that epigenetic silencing was responsible for lower levels of *DUSP6* transcription [23]. Overall this data was interpreted as evidence that *DUSP6*/MKP-3 is associated with progression from early PanINs to invasive ductal adenocarcinoma (PDAC). Similar data has also implicated *DUSP6*/MKP-3 as a tumour suppressor in mutant *Kras*-driven lung tumours, where expression levels showed an inverse relationship with both growth activity and histological grade. Loss of heterozygosity of the *DUSP6* locus was also found in 17.7% of lung cancer cases and was associated with reduced expression levels [25].

*In vitro* studies seemingly support this interpretation; ectopic expression of *DUSP6*/MKP-3 in either pancreatic or lung cancer cells resulted in the suppression of cell growth and apoptosis [22], [25], while expression of *DUSP6*/MKP-3 in *Hras* transformed fibroblasts caused a significant delay in tumour formation after injection into nude mice [26]. However, the latter studies should be treated with considerable caution as overexpression of *DUSP6*/MKP-3 using constitutively active heterologous promoters may result in complete or near complete ablation of ERK activity. As ERK activation is known to be required for S-phase entry and cell proliferation [27], [28], it is possible that the ectopic expression of non-physiological levels of *DUSP6*/MKP-3 may result in artefactual suppression of tumour cell proliferation and growth. Clearly, mouse models of Ras and Braf-induced cancers coupled with *DUSP6* gene knockout and a more rigorous assessment of *DUSP6*/MKP-3 levels in larger cohorts of human tumours will be instrumental in clarifying the possible role of *DUSP6*/MKP-3 as a tumour suppressor.

### 2.2 DUSP6/MKP-3 may be pro-oncogenic in certain tumour types

While the work outlined above suggests a tumour suppressor role for *DUSP6*/MKP-3, other studies have indicated that this phosphatase may be oncogenic. *DUSP6*/MKP-3 is upregulated in human glioblastoma cell lines. Overexpression of *DUSP6*/MKP-3 in these cells led to the expected reduction in proliferation rate, but also affected cell morphology with *DUSP6*/MKP-3-overexpressing cells exhibiting a more flattened appearance, lower levels of cellular detachment after stimulation with EGF and an increased propensity to form colonies in soft agar. Surprisingly, mouse xenograft experiments showed that tumours arising from glioblastoma cells expressing *DUSP6*/MKP-3 grew significantly faster than non-expressing controls perhaps reflecting these changes in cell adhesion and morphology [29]. Overexpression of *DUSP6* has also been identified in a subset of mouse melanoma cell lines, where it is associated with enhanced anchorage-independent growth and invasive capacity [30] and overexpression of *DUSP6*/MKP-3 in papillary thyroid carcinoma (PTC) cell lines is associated with increased cell migration and invasion [31]. However, perhaps the most persuasive data implicating *DUSP6*/MKP-3 as a pro-oncogenic phosphatase has come from a recent study of pre-B cell transformation in acute lymphoblastoid leukemia (ALL) [32].

Müschen and colleagues noted that the acute activation of oncogenes such as BCR-Abl or *Nras*^G12D^ in human pre-B cells invariably led to cell death. However, the small fraction of cells that survived and became transformed all exhibited increased expression of negative regulators of ERK signalling including *DUSP6*/MKP-3, the transcription factor ERM (Ets related molecule, also known as Ets Variant Gene 5, ETV5) and sprouty-2 (Spry-2). These findings were extended to pre-B ALL cells, which unlike normal pre-B cells, were also primed to express negative regulators of the ERK pathway. This up regulation was dependent on both BCR-Abl and ERK activity as evidenced by sensitivity to a tyrosine kinase inhibitor (TKI) (Imatinib) or MEK inhibitor (PD325901). Interestingly, *DUSP6* mRNA levels were a robust and independent predictor of outcome for adults with Philadelphia chromosome positive (*Ph^+^*) (BRC-Abl-driven) ALL, with higher than median *DUSP6* mRNA levels correlating with shorter overall survival [32]. To explore the specific relevance of *DUSP6*/MKP-3, B cell lineage and myeloid progenitor cells obtained from the bone marrow of *DUSP6*^−/−^ mice and wild-type controls were transformed with BCR-Abl1. Interestingly, while the myeloid progenitors transformed with BCR-Abl1 showed a higher colony forming ability in the absence of *DUSP6*/MKP-3, indicating a tumour suppressor function, the colony formation of *DUSP6*^−/−^ B cell lineage leukaemia was significantly reduced, consistent with a positive role for this phosphatase in malignant transformation. In a series of further experiments using conditional expression of *Nras*^G12D^ in pre-B cells from wild type and *DUSP6*^−/−^ mice, only cells from the wild type mice were susceptible to transformation and shRNA-mediated knockdown of *DUSP6* significantly reduced the colony forming ability of human pre-B ALL cells. Furthermore, the growth of pre-B cells transduced with BCR-Abl in the presence of Imatinib was strictly dependent on *DUSP6* after washing out the inhibitor, again showing that pre-B ALL cells are dependent on robust *DUSP6*/MKP-3-mediated negative feedback control of ERK signalling for continued growth and survival [32].

Perhaps the most provocative series of experiments presented in this study involve the use of a pharmacological inhibitor of *DUSP6*/MKP-3 activity to validate the hypothesis that this phosphatase might be a therapeutic target in human ALL. BCI (2-benzylidene-3-(cyclohexylamino)-1-Indanone hydrochloride) was first identified in a screen for compounds which were able to increase FGF signalling output in Zebrafish embryos and *DUSP6*/MKP-3 was identified as the relevant biological target. Biochemical studies indicated that BCI specifically inhibits *DUSP6*/MKP-3 by preventing the ERK-dependent allosteric changes that occur within the active site, thus preventing catalytic activation [33]. In support of this, BCI caused a rapid increase in ERK activity in patient-derived *Ph^+^* ALL cells. Furthermore, the increased ERK signalling was associated with increased levels of intracellular reactive oxygen species (ROS) and p53-mediated cell death. When ALL cells with hyperactive ERK were chronically treated with MEK inhibitor they adapted to normal growth in its presence. However, washout of the drug caused a subsequent “rebound” of ERK activity and sensitised cells to treatment with BCI by a factor of approximately 6. This strongly suggests that there is a threshold of ERK-signaling that has to be overcome to trigger BCI-induced apoptosis. Finally, mouse xenograft experiments using *Ph^+^* ALL cells derived from patients after relapse during ongoing therapy with tyrosine kinase inhibitors showed these to be insensitive to Imatinib, but significantly sensitive to treatment with BCI, indicating that this drug may be of utility in treating TKI-resistant *Ph^+^* ALL [32].

These experiments demonstrate a lineage specific effect of *DUSP6*/MKP-3, with the phosphatase acting as a tumour suppressor in myeloid cells, but as an essential mediator of malignancy in pre-B cells and suggest that this undue reliance on negative feedback regulators of ERK activity may present a vulnerability and reveal novel drug targets in pre-B cell malignancy. However, some caution must be exercised in the interpretation of the experiments using BCI. Although active against *DUSP6*/MKP-3, this drug is acknowledged to be both non-specific with respect to MKPs and is also relatively toxic. As deletion of *DUSP6*/MKP-3 is tolerated in the mouse [34], the whole organism and cellular toxicity exhibited by BCI is highly likely to reflect off-target effects. This interpretation is also supported by the observed inhibition of PI3-kinase-akt signalling, coupled with a global reduction in levels of cellular phosphotyrosine observed in the BCI-treated *Ph^+^* ALL cells. BCI contains an electrophilic α,β-unsaturated ketone moiety, which is often viewed as a liability in drug development due to non-selective modification of cellular nucleophiles. This is acknowledged in a recent publication in which derivatives of BCI have been synthesised in an attempt to reduce this toxicity while retaining activity towards *DUSP6*/MKP-3 [35]. It will be interesting to determine if these derivatives, several of which show equivalent or higher potency as inhibitors of *DUSP6*/MKP3, but are considerably less toxic, can also selectively kill transformed pre-B ALL cells.

### 2.3 DUSP5: a nuclear ERK phosphatase and tumour suppressor

*DUSP5* is one of four closely related inducible nuclear MKPs. However, it is unique within this subgroup that unlike *DUSP1*/MKP-1, *DUSP2* or *DUSP4*/MKP-2 it is devoid of activity towards the stress activated MAPKs, acting as an ERK selective phosphatase [36]. Furthermore, transcriptional induction of *DUSP5* in response to growth factor stimulation is ERK dependent and it can also bind and sequester inactive ERK in the nucleus when expressed in mammalian cells [36], [37]. Thus, *DUSP5* can be regarded as the nuclear counterpart of *DUSP6*/MKP-3 in regulating the spatiotemporal activity of the Ras/ERK pathway.

As mentioned previously, both *DUSP5* and *DUSP6*/MKP-3 are amongst a subset of genes, which are often upregulated in tumours and cancer cell lines in which Ras/MAPK signalling is activated. Loss of *DUSP5* expression has been detected in advanced gastric and prostate cancers, where its loss correlates with a poor patient outcome. Furthermore, the re-expression of *DUSP5* in gastric cancer cell lines reduced both cell proliferation and colony forming ability *in vitro* [38], [39]. While these limited studies indicate that *DUSP5* might act as a tumour suppressor, they rely on correlation between expression level and clinical outcome coupled with over expression of *DUSP5 in vitro*. However, a recent study using a combination of genetic and biochemical studies has now provided the first evidence of a *bona fide* tumour suppressor function for *DUSP5* [40].

In order to dissect the role of *DUSP5* in Ras-induced tumourigenesis, mice were generated in which the *DUSP5* gene was deleted by homologous recombination and found to be both viable and fertile. Animals were then studied using the well-established DMBA/TPA-inducible multi-stage skin carcinogenesis protocol in which DMBA-induced *Hras* mutations at codon 61 (*Hras*^Q61L^) drive skin papilloma induction. Mice lacking DUSP5 developed twice as many skin tumours when compared with wild type animals, while mice lacking one copy of *DUSP5* showed an intermediate phenotype. Loss of *DUSP5* did not influence the mechanism of carcinogenesis as 90% of the *DUSP5*^−/−^ papillomas contained the signature *Hras*^Q61L^ mutation, nor was tumour morphology or size affected by *DUSP5* deletion. In searching for a mechanistic basis for this increased tumourigenesis, the spatiotemporal regulation of ERK signalling was studied using high-content microscopy, which allowed both the visualisation and quantification of levels of nuclear and cytoplasmic phospho-ERK (*p*-ERK) and total ERK in TPA-treated mouse embryo fibroblasts (MEFs). These studies revealed two major effects of *DUSP5* loss in these cells. Firstly, levels of nuclear *p*-ERK were significantly higher at early times after TPA stimulation in *DUSP5*^−/−^ cells compared to wild type MEFs, a result also confirmed using biochemical cell fractionation. Secondly, levels of total ERK were much lower in the knockout MEFs at later times after stimulation. Importantly, both phenotypes could be reversed by the adenoviral expression of wild-type *DUSP5*, but not a KIM mutant (*DUSP5*^R53/54A^), expressed under the control of the early growth response 1 (Egr1) promoter.

**Fig. 2:**
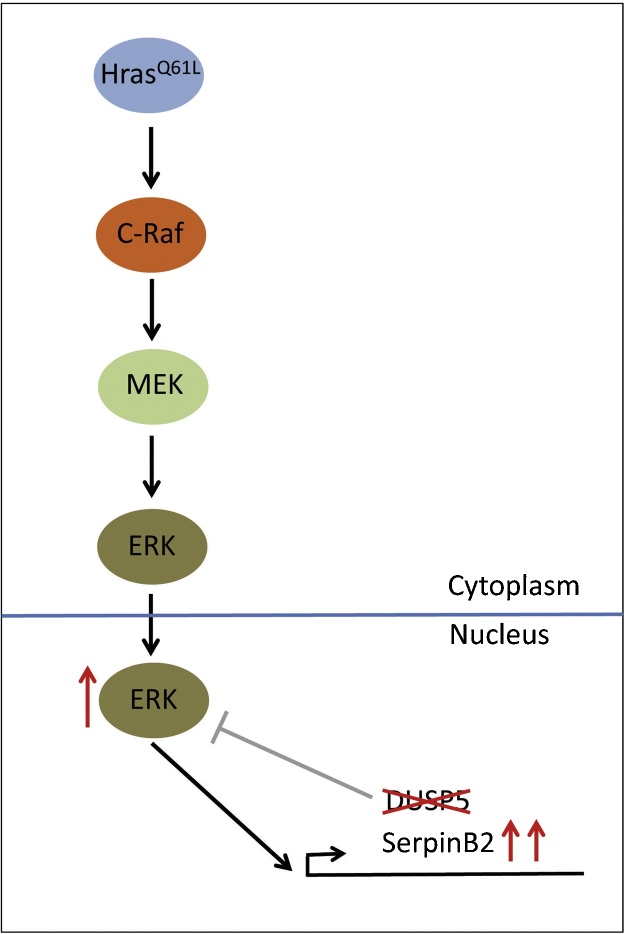
Loss of the inducible ERK-specific phosphatase encoded by *DUSP5* specifically affects nuclear ERK signalling and gene expression. Loss of *DUSP5* synergises with mutant *Hras*^Q61L^ leading to higher levels of nuclear *p*-ERK. This selectively up-regulates a small number of ERK target genes, including the gene encoding serpinB2. It is the increased expression of the latter protein that leads to the increased sensitivity to DMBA/TPA induced skin papillomas in *DUSP5* knockout mice.

Microarray experiments in TPA-treated MEFs revealed that *DUSP5* loss caused the increased expression of a small subset of TPA-inducible ERK-dependent genes and that *serpinB2* (also known as plasminogen activator inhibitor 2, *PAI2*) was expressed at the highest level after *DUSP5* deletion. Furthermore, *DUSP5* loss synergises with mutant *Hras*^Q61L^ in driving ERK-dependent *serpinB2* expression in TPA treated MEFs (Fig. 2). Interestingly, *serpinB2* had previously been identified as a promoter of DMBA–TPA carcinogenesis when expressed in the skin of transgenic mice under the control of the bovine keratin 5 promoter [41] and combining *DUSP5* deletion with loss of *serpinB2* completely reversed the sensitivity to carcinogenesis caused by *DUSP5* loss. Overall this work demonstrates that *DUSP5* has an essential non-redundant function in regulating both the activity and localisation of nuclear ERK signalling and that *DUSP5* also acts as a tumour suppressor to limit the oncogenic potential of mutant *Hras*^Q61L^ in this cancer model [40]. At present the precise mechanism by which increased *serpinB2* expression promotes tumour development is unclear, but it may involve intracellular functions, rather than its canonical role as a secreted inhibitor of extracellular proteases [42].

It will be very interesting to determine if *DUSP5* plays a wider role as a tumour suppressor in more clinically relevant mouse models of *Hras* or *Braf*-driven cancers such as pancreas, lung or intestinal tumours and to determine the extent to which it is deregulated in a wider range of human cancers. Interestingly, mice lacking *DUSP5* also display prolonged eosinophil survival and enhanced eosinophil effector functions following experimental helminth infection. Microarray experiments in interleukin-33 (IL-33) stimulated wild type and *DUSP5*^−/−^ eosinophils also revealed changes in ERK-dependent gene expression caused by DUSP5 loss [43]. Experiments looking at the expression of genes involved in the regulation of cell death revealed a novel mechanism by which *DUSP5* regulates eosinophil survival through increased ERK-dependent expression of the anti-apoptotic BL2 family member BCL-X_L_. This indicates that the effects of *DUSP5* deletion on gene expression may be lineage and/or stimulus specific. It is therefore possible that *DUSP5* loss may affect oncogenic ERK signalling differently, depending on the tissue and cellular context.

## 3 ERK-specific MKPs and sensitivity to anti-cancer drugs

In addition to their roles in the genesis and development of cancer it has long been appreciated that MAPK signalling is an important determinant of cell and tissue responses to many physical and chemical agents used in cancer therapy. Thus MKPs, as regulators of MAPK activity, have also been implicated in modulating cancer cell/tumour sensitivity to both chemotherapeutic drugs and radiation. Probably the most widely studied of the MKPs in this regard is *DUSP1*/MKP-1, an inducible nuclear MKP, which shows activity towards all three major MAPKs in the rank order JNK > p38 = ERK [44]. *DUSP1*/MKP-1 has been implicated in acquired or intrinsic resistance to a wide range of anti tumour drugs including cisplatin, taxanes, anthracyclins and doxorubicin. In most of these cases, resistance has been linked with the increased expression of *DUSP1*/MKP-1 and its substrate preference for JNK, which is a well-established positive regulator of apoptotic cell death [11]. In contrast, the role of ERK-specific phosphatases has been less well defined. However, recent publications have indicated that these enzymes may play a more complex role in mediating drug resistance in human lung cancers with their drug-induced down regulation facilitating the re-activation of Ras/ERK signalling in treated cells, thus aiding cell survival and promoting resistance to therapy.

### 3.1 Modulation of DUSP6/MKP-3 levels modifies the response to targeted therapies in lung cancer

Non-small cell lung cancer (NSCLC) is the most prevalent form of this disease accounting for approximately 85% of cases. Of these, a significant fraction (∼15%) contain activating mutations in the epidermal growth factor receptor (EGFR) making the development and use of EGFR tyrosine kinase inhibitors (TKIs) a focus of drug therapy in this disease [45], [46]. However, despite the efficacy of these drugs in target inhibition, only a very small fraction of patients (∼5%) achieve a near complete tumour response and it has been speculated that a portion of these non-responders may have intrinsic, rather than acquired, resistance to TKIs [47]. A recent study has explored these resistance mechanisms by dissecting out the molecular machinery by which the Ras/ERK pathway is activated after EGFR inhibition in NSCLC cells [48].

Using cancer cell lines that express mutant forms of EGFR, it was demonstrated that exposure to the TKI gefitinib (Iressa) caused cell death in approximately 95% of treated cells. However, the surviving cells were both resistant to the drug and capable of both proliferating and forming colonies *in vitro*, a result consistent with a mechanism of innate resistance. Biochemical analysis of these cells revealed that although TKI treatment effectively blocked EGFR activation, it only caused a transient blockade of downstream ERK activation, with a marked rebound in ERK activity in the continued presence of the drug. This TKI-induced ERK reactivation was supressed by a MEK inhibitor and Ras activity was also essential, as evidenced by its sensitivity to a dominant negative mutant of *Kras* (*Kras*^S17N^). Surprisingly, the continued activity of the Ras/ERK pathway was not due to the activation of non-EGFR RTKs or the Src tyrosine kinase in these cells [48], the latter of which is known to cause activation of the Ras/MAPK pathway in cells harbouring mutant EGFR [49], indicating that ERK must be reactivated by an additional (as yet undefined) mechanism.

**Fig. 3:**
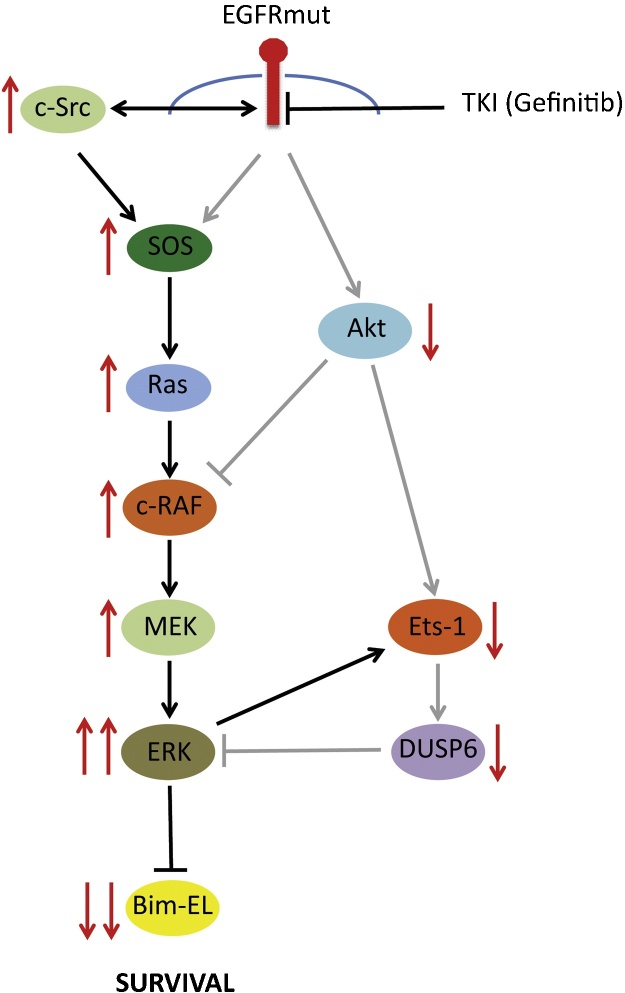
In NSCLC cells harbouring activating mutations in the epidermal growth factor receptor (EGFR) and treated with the tyrosine kinase inhibitor (TKI) Gefitinib, Ras/ERK and PI3-kinase-Akt signalling are inhibited. However, while Akt activity remains low, Ras/ERK pathway activity recovers. This recovery is driven in part by the activity of the Src tyrosine kinase, but also by the loss of expression of the ERK-specific phosphatase *DUSP6*/MKP-3. Loss of *DUSP6* expression is secondary to the continued inhibition of Akt activity by Gefinitib, resulting in lower levels of the transcription factor Ets1, which normally transactivates the *DUSP6* gene promoter to increase expression. This elevated Ras/ERK pathway activity contributes to TKI resistance by reducing the level of the pro-apoptotic Bcl2 family protein Bim-EL, thus promoting cell survival in the continued presence of the drug.

A survey of the expression profiles of protein phosphatases in cells exposed to another EGFR TKI erlotinib (Tarceva) revealed that the drug caused a significant reduction in the expression of *DUSP6*/MKP-3, a result confirmed at the mRNA and protein level in EGFR mutant NSCLC cells in the presence of gefitinib. Furthermore, adenoviral expression of *DUSP6* in NSCLC cells in the presence of TKI blocked the reactivation of ERK despite the continued activation of Ras and MEK. The loss of *DUSP6* expression was secondary to loss of the transcription factor Ets1, which is known to regulate ERK-dependent *DUSP6/*MKP-3 transcription in both fibroblasts and lung cancer cells [17], [18]. In an unexpected twist, it was shown that the loss of Ets1 expression was due to TKI-mediated inhibition of PI3-kinase signalling, rather than any change in ERK activity and that *DUSP6* expression could be rescued by expression of a constitutively active form of Akt. Finally, the mechanism by which the elevated ERK activity engendered by TKI treatment of NSCLC cells enhances survival was identified as the increased ERK-dependent phosphorylation of the extra long isoform of the pro-apoptotic BH3 only Bcl-2 family member Bim (Bim EL) (Fig. 3). This promotes its proteosomal degradation and thus blocks cell death. This study shows an unexpected convergence of ERK and Akt signalling in the regulation of Ets1 and its target genes including *DUSP6*/MKP-3 and demonstrates that loss of *DUSP6*/MKP-3 plays a key role in reinforcing the increased ERK signalling that is coupled to cell survival in the face of specific EGFR inhibitors in NSCLC [48].

In addition to EGFR mutations, a significant fraction of NSCLC is caused by expression of a fusion between echinoderm microtubule-associated protein-like 4 (ELM4) and the RTK anaplastic lymphoma kinase (ALK) [50]. These ELM4-ALK positive tumours, like those with mutations in EGFR, either show a lack of response to ALK-specific TKIs such as crizotinib (Xalkori) or relapse with drug resistant disease [51]. In an attempt to identify molecular mechanisms, which might limit the effectiveness of ALK inhibitors, Hrustranovic et al. [52] studied the effect of inhibiting downstream signalling pathways in ELM4-ALK lung adenocarcinoma cells. They found that inhibitors of ERK signalling were equally as effective as ALK inhibitors in causing decreased cell growth. In contrast, constitutive activation of Ras/ERK signalling enabled cells to survive and even proliferate in the presence of crizotinib, thus revealing a specific requirement for ERK signalling in these lung adenocarcinoma cells. How ELM4–ALK actually engages the Ras/ERK pathway was unclear, particularly as the fusion protein lacks the transmembrane domain within ALK that would normally anchor the RTK within the plasma membrane and facilitate interactions with downstream effectors. In this regard they investigated a unique hydrophobic echinoderm microtubule-associated protein-like protein (HELP) domain within ELM4, reasoning that this might facilitate membrane association of the ELM4–ALK fusion and facilitate downstream signalling to ERK. In agreement with this hypothesis, wild type ELM4–ALK protein localised to discrete intracellular compartments and activated Ras/ERK signalling, while a mutant lacking the HELP domain showed diffuse staining and failed to activate ERK [52]. These findings led to the suggestion that ALK inhibitors combined with a sub maximal dose of a MEK inhibitor might be more effective than a TKI such as crizotinib alone. This was verified using both *in vitro* and *in vivo* (mouse xenograft) experiments in which the combined drug regimen was far more effective in blocking both ERK activity and tumour growth when compared with treatment using either drug alone.

On the basis of these findings they also hypothesised that acquired resistance to ALK-specific TKIs was highly likely to involve reactivation of the Ras/ERK pathway and this was confirmed in experiments in which ELM4–ALK positive lung cancer cells were rendered drug resistant by chronic exposure to crizotinib. In a series of experiments to determine the likely mechanism of this resistance, exome sequencing revealed an increased copy number for the gene encoding wild type *Kras* in one line of crizotinib resistant cells and in a study of tumour biopsies taken from patients with acquired resistance to TKI therapy 3 out of 15 patients (20%) had focal amplification of *Kras* indicating that this is a *bona fide* resistance mechanism to ALK-specific TKIs in lung adenocarcinoma. However, other TKI resistant cell lines had relatively low Ras activity, but still displayed high basal ERK activation, indicating that other mechanisms are at play in the rescue of Ras/ERK activity in these cells [52]. A survey of dual-specificity MKP expression in parental and drug resistant ELM4–ALK lung cancer cells revealed that while the parental cells had high levels of *DUSP6* mRNA expression the drug resistant cells consistently had lower levels of this phosphatase. Stable reconstitution of *DUSP6*/MKP-3 restored both sensitivity to crizotinib and the ability of this drug to supress ERK signalling while shRNA-mediated knockdown of *DUSP6*/MKP-3 promoted crizotinib resistance in parental ELM4–ALK lung adenocarcinoma cells and this was accompanied by rescue of ERK activation. Finally, in a survey of either drug naïve (*n* = 15) or drug resistant (*n* = 10) ELM4–ALK patient tumours *DUSP6*/MKP-3 protein levels as detected and scored by immunohistochemical staining were significantly lower in the drug resistant samples, while in 5 out of 6 (80%) of paired tumour biopsies from patients taken either before or after drug treatment *DUSP6* expression was lower in the resistant samples. Taken together, these biochemical and clinical studies strongly implicate the down regulation of *DUSP6*/MKP-3 as a driver of ERK-dependent resistance to TKI therapy in ELM4–ALK positive lung adenocarcinomas [52].

Either mutations in EGFR or expression of the ELM4–ALK fusion drive tumourigenesis in almost 20% of NSCLC cases worldwide. In both cases either acquired or intrinsic resistance to EGFR or ALK-targeted TKIs is driven by reactivation of the Ras/ERK signalling pathway and a major factor in this reactivation and the genesis of drug resistance is loss of *DUSP6*/MKP-3. In the case of EGFR mutant tumours, *DUSP6*/MKP-3 loss appears secondary to down regulation of the transcription factor and ERK signalling target Ets1, while the mechanism of *DUSP6*/MKP-3 down regulation in ELM4–ALK tumour cells was not studied. It will be interesting to see if similar mechanisms operate in other tumour types and there are indications that this may well be the case. For instance, the prolonged use of MET (hepatocyte growth factor receptor [HGFR]) RTK inhibitors in gastric cancer cell lines *in vitro* results in loss of *DUSP6*/MKP-3 expression, which correlates with increased levels of *p*-ERK [53] while loss of *DUSP6*/MKP-3 in ovarian cancer has also been linked with cisplatin resistance [54].

## 4 Conclusions and remarks

From the experimental evidence reviewed above it is now clear that dual-specificity MAP kinase phosphatases play a key role in the regulation and outcome of oncogenic signalling through the Ras/ERK pathway. From studies of the inducible nuclear phosphatase *DUSP5*, it is also clear that these effects can reflect not only their activity towards the ERK1/2 MAP kinases but also their spatial localisation. Perhaps not surprisingly, individual MKPs can exhibit either tumour suppressor function or can act as pro-oncogenic regulators of Ras/ERK signalling and this may depend on the differing and tissue-specific thresholds of ERK activity that are either permissive for or promote cell proliferation or conversely cause cell cycle arrest/senescence or cell death. Obviously, where tumour suppressor functions for MKPs are manifest, then specific inhibition is to be avoided. However, in those malignancies in which MKPs have a proven role in promoting tumour growth, then such inhibitors may be of therapeutic use. As a class of enzymes, cysteine-dependent protein tyrosine phosphatases, of which the MKPs are a subfamily, have been amongst the most difficult targets against which to develop specific inhibitors [55]. However, the emergence of chemical entities, which target the allosteric activation of MKPs, which occurs on MAPK binding, rather than the active site itself, offer the promise that more selective and less toxic agents can be developed and tested. Finally, studies of the regulation and role of *DUSP6*/MKP-3 in cellular responses to RTK inhibitors have revealed that loss of function may play a key role in resistance to these drugs. This reinforces the importance of signalling plasticity and pathway remodelling in the emergence of drug resistance and it is perhaps no surprise that MKPs, as major regulators of MAPK activity, are key players in this process.
